# Supplementary material for: Inhibitory Activity of Essential Oils against Vibrio campbellii and Vibrio parahaemolyticus
Source: Microorganisms. 2020 Dec 8;8(12):1946. doi: 10.3390/microorganisms8121946 (PMC7763747; doi:10.3390/microorganisms8121946)
Supplement: Supplementary file 1 [file microorganisms-08-01946-s001.pdf]

Supplementary information

Table S1: Twenty-two EOs used in this study with their components (≥10%) and assigned chemical class of components. When no EOCs present at > 10% (n=2): only EOC at highest concentration is shown [15]. ct=chemotype; #=organic EO

Essential oil of plant species	Part of plant	Major components (≥10% v/v)	%	Chemical class
Abies alba	needles	α-PINENE	34.30	monoterpenes
		LIMONENE	19.42	monoterpenes
		β-PINENE	17.71	monoterpenes
		CAMPHENE	12.90	monoterpenes
Apium graveolens	fruits	LIMONENE	63.69	monoterpenes
		β-SELINENE	17.55	sesquiterpenes
Artamisia dracunculus	flowering tops	ESTRAGOLE	79.36	phenol methyl ethers
Artemisia herba alba	flowering tops	α-THUJONE	61.45	ketones
		CAMPHOR	12.47	ketones
		β-THUJONE	10.39	ketones
Cinnamomum camphora ct linalool	wood	LINALOOL	98.35	monoterpenols
Cinnamomum cassia	twigs	CINNAMALDEHYDE	78.45	aldehydes
		trans-p-METHOXY-CINNAMALDEHYDE	10.75	phenol methyl ethers
Cinnamomum zeylanicum	leaves	EUGENOL	74.35	phenol methyl ethers
Citrus sinensis	peels	LIMONENE	95.10	monoterpenes
Cuminum cyminum	fruits	CUMINAL	31.94	aldehydes
		γ-TERPINENE	18.32	monoterpenes
		β-PINENE	16.71	monoterpenes
		p-CYMENE	16.31	monoterpenes
Curcuma longa[#]	roots	ar-TURMERONE	38.76	ketones

		α-TURMERONE	23.37	ketones
Cymbopogon martini variety motia	aerial parts	GERANIOL	78.99	monoterpenols
Eucalyptus citriodora ct citronellal[#]	leaves	CITRONELLAL	80.02	aldehydes
Eucalyptus dives ct piperitone[#]	leaves	PIPERITONE + BICYCLOGERMACRENE	39.04	ketones+ sesquiterpenes
		α-PHELLANDRENE	22.20	monoterpenes
		1,8-CINEOLE + β -PHELLANDRENE	10.79	ethers+ monoterpenes
Laurus nobilis	leaves	1,8-CINEOLE	41.4	ethers
Litsea citrata	fruits	CITRAL	71.35	aldehydes
		LIMONENE	11.53	monoterpenes
Melaleuca alternifolia	leaves	TERPINENE-4-OL	41.35	monoterpenols
		γ-TERPINENE	20.64	monoterpenes
Mentha × piperita	aerial parts	MENTHOL	37.61	monoterpenols
		MENTHONE	23.98	ketones
Mentha pulegium	aerial parts	PULEGONE	84.14	ketones
Petroselinum crispum	leaves	1,3,8-p-MENTHATRIENE	20.75	monoterpenes
		α-PINENE	18.76	monoterpenes
		MYRISTICINE	13.64	phenol methyl ethers
		β-PINENE	12.74	monoterpenes
Pogostemon cablin	flowering tops	PATCHOULOL	30.15	sesquiterpenols
		α-BULNESENE	18.00	sesquiterpenes
		α-GUAIENE	14.98	sesquiterpenes
Thymus zygis	flowering tops	THYMOL	48.13	phenols
		p-CYMENE	21.22	monoterpenes
Zingiber officinalis[#]	rhizome	α-ZINGIBERENE	24.00	sesquiterpenes
		β-SESQUIPHELLANDRENE	10.85	sesquiterpenes
		CAMPHENE	10.34	monoterpenes

Table S2: Highly enriched EOCs used in this study with their purity and assigned chemical class [15].

**Essential oil component**	**Purity (%)**	**Chemical class**
(-)-TERPINEN-4-OL	⩾95	monoterpenols
(-)-β-PINENE	99	monoterpenes
(+)-CARVONE	⩾98.5	ketones
(±)-CINTRONELLAL	⩾95	aldehydes
4-ALLYLANISOLE	98	phenol methyl ethers
CINNAMALDEHYDE	99	aldehydes
CITRAL	95	aldehydes
EUGENOL	99	phenol methyl ethers
GERANIOL	98	monoterpenols
R-(+)-LIMONENE	97-98	monoterpenes
S-(-)-LIMONENE	⩾95	monoterpenes
α-PINENE	98	monoterpenes

Table S3: Summary of vapour-phase-mediated susceptibility assay (VMS assay), bacterial growth assay and specific quorum sensing-inhibitory assay of essential oils.

**Essential oil of plant species**	**VMS assay**		**Bacterial growth assay**		**Specific quorum sensing-inhibitory assay**	
	Category				A_{QSI}, 0.001%	A_{QSI}, 0.0001%
	iVMAA >3	$iVMAA_{90}$ >3	Fold (0.001%) <0.5	Fold (0.0001%) <0.5	>2	>2
Artemisia herba alba	+	+	-	-	-	-
Cinnamomum camphora ct linalool	+	+	-	-	-	-
Cinnamomum cassia	-	-	+	+	-	-
Cuminum cyminum	-	-	-	-	+	-
Eucalyptus citriodora ct citronellal[#]	+	+	-	-	+	-
Litsea citrata	+	+	+	+	-	-
Melaleuca alternifolia	+	+	+	+	-	-
Mentha pulegium	-	-	+	-	+	-
Zingiber officinalis[#]	-	-	-	-	+	-

iVMAA > 3: the category of inhibitory vapour-phase-mediated antimicrobial activity was higher than 3, $iVMAA_{90}$ > 3: the category of 90% reduction of growth (compared to the control growth) was higher than 3, Fold (0.001%) < 0.5: the fold (compared to control) of OD value was lower than 0.5 with the concentration of 0.001%, Fold (0.0001%) < 0.5: the fold (compared to control) of OD value was lower than 0.5 with the concentration of 0.0001%, A_{QSI}, 0.001% > 2: specific quorum sensing inhibitory activity is higher than 2 with the concentration of 0.001%, +: positive detection, -: negative detection.
